# Detection of an antibody against *Plasmodium vivax *in residents of Gimpo-si, South Korea, using an indirect fluorescent antibody test

**DOI:** 10.1186/1475-2875-10-19

**Published:** 2011-01-31

**Authors:** Won-Ja Lee, Hyung-Hwan Kim, Soon-Mi Hwang, Mi-Young Park, Nam-Ryul Kim, Shin-Hyeong Cho, Tae-Sook In, Jung-Yeon Kim, Jetsumon Sattabongkot, Youngjoo Sohn, Hyuck Kim, Jong-Koo Lee, Hyeong-Woo Lee

**Affiliations:** 1 Division of Malaria and Parasitic Diseases, National Institute of Health, Korea Centers for Disease Control and Prevention, Cheongwon-gun 363-951, Republic of Korea; 2 Vascular Medicine Research Unit, Brigham and Women's Hospital, Harvard Medical School, Cambridge, MA 02139, USA; 3 Public Health Center, Gimpo 415-730, Republic of Korea; 4 Department of Entomology, Armed Forces Research Institute of Medical Sciences, Bangkok 10400, Thailand; 5 Department of Gynecology, College of Oriental Medicine, Sangji University, Wonju 220-717, Republic of Korea; 6 International Research Center for Bioscience and Biotechnology, Jungwon University, Goesan 367-805, Republic of Korea; 7 Korea Centers for Disease Control and Prevention, Ministry of Health & Welfare Seoul 122-701, Republic of Korea; 8 Department of Pathology, University of Florida, J-566, 1600 SW Archer Road, Gainesville, FL 32610, USA

## Abstract

**Background:**

First reemerged malaria case was reported in 1993 after two decades absent in South Korea. Thereafter, *Plasmodium vivax *spreads out near demilitarized zone (DMZ). This study investigated the prevalence of *P. vivax *after the malaria transmission season in Gimpo-si where adjacent to DMZ of South Korea. An indirect fluorescent antibody test (IFAT) was performed to evaluate anti-malaria antibodies in blood samples.

**Methods:**

Microscopic examinations were performed to identify the presence of malaria parasites. Antibodies against *P. vivax *were detected using IFAT, and blood samples from antibody-positive cases were tested using a polymerase chain reaction (PCR) assay that detects malaria parasites.

**Results:**

A total of 5,797 blood samples were collected from residents in Gimpo-si. The positivity rate by IFAT was 2.16% (n = 125). Yangchon-myeon (3.28%) had the highest positivity rate of the seven administrative districts tested. Positivity rates increased with age (*P *< 0.05). Sixteen of the IFAT positive samples (12.80%, n = 125) were positive for malaria DNA according to PCR. Blood samples with an antibody titer over 1:256 had high positivity rates in the PCR analysis (*P *< 0.05).

**Conclusions:**

These results indicate that antibody titers obtained using IFAT may provide useful information about the prevalence of *P. vivax *in low endemic areas and could be used to detect asymptomatic patients. Finding asymptomatic patients is important in eliminating vivax malaria in South Korea.

## Background

*Plasmodium vivax*, a causative agent of relapsing benign tertian human malaria, is the second- most important human malaria and afflicts several hundred million people annually. This disease is a major public health problem, with associated socioeconomic ramifications, for many temperate and most tropical countries [1].

For several centuries, vivax malaria has been prevalent throughout the Korean peninsula. The first scientific documentation of malaria was published in 1913. At that time, malaria occurred throughout the country without noticeable geographical distinctions [2]. However, the incidence of vivax malaria has rapidly decreased [3,4] due to a national malaria eradication program and help from the World Health Organization (WHO). Vivax malaria was believed to have been eradicated in South Korea in the late 1970s, although two sporadic cases were detected in the 1980s. These two patients relapsed after a long incubation period [5]. In 1993, a soldier in the South Korea army serving in northern Gyeonggi-do was diagnosed with vivax malaria [6]. Subsequently, Cho *et al *reported two civilian patients infected with vivax malaria [7]. Thereafter, a total of 2,198 vivax malaria patients were detected between 1994 and 1997 near the demilitarized zone (DMZ), centering around the villages of Paju-si, Yeoncheon-gun, Cheorwon-gun, Gimpo-si, Ganghwa-gun, Goyang-si, and Dongducheon-si. There is a great concerned that this re-emergence may lead to the re-establishment and geographical expansion of malaria [8]. The north-western region of Gyeonggi-do has been ecologically preserved because people are not allowed in the DMZ [9]. These factors raise the possibility that vivax malaria has been reintroduced and that there is active local transmission.

The rainy season usually begins in late June and extends through the end of July. In South Korea, the potential vectors of vivax malaria are *Anopheles sinensis, Anopheles kleini*, and *Anopheles pullus*. Malaria transmission is expected to begin 3-4 weeks after the appearance of vector mosquitoes in mid-May. *An. sinensis *(63.3%) was the most abundant anopheline mosquito captured in malaria high-risk areas (northern Gyeonggi-do) near the DMZ in South Korea, followed by *An. kleini *(24.7%) and *An. pullus *(8.7%) [10]. The incidence of malaria peaks in August after the rainy season and declines to baseline levels during mid-October. Therefore, blood collection was carried out between late October and mid-December, when the anopheline population disappears.

Gimpo-si is located in north-western South Korea and is surrounded by the Imjin River and the Han River, which are close to North Korea. The Korean National Institute of Health (KNIH) reported one case of malaria in 1995, one in 1996, 15 in 1997, and 65 in 1998. These cases led the malaria research team at the KNIH to investigate the prevalence of malaria in Gimpo-si using microscopy and indirect fluorescent antibody tests (IFATs). Seroimmunological diagnosis, in particular by IFAT, is an important tool for the detection of malaria, especially when microscopic evidence of the parasites is not available due to the several reasons [11-14]. Blood samples were collected from 845 residents in this area from November to December 1998. Twenty-four residents were seropositive for malaria by IFAT. Four seropositive residents (16.7%) developed symptoms of malaria in the following year. Based on the results from these preliminary trials, there is ample evidence to suggest that there are asymptomatic malaria patients in this area. In this study, the significant role that IFAT plays in identifying asymptomatic patients and providing information on the prevalence of malaria is demonstrated.

## Methods

### Study areas and blood sample collection

The study was conducted in Gimpo-si, Gyeonggi-do, South Korea, from late October to mid- December 1999. All participants were adult volunteers who were enrolled after the nature of the study was explained and verbal informed consent was obtained. Approximately 3 ml of blood was collected from each individual. Thin and thick blood smears were prepared for microscopic examination (magnification 7 × 100). The blood samples were transferred to the KNIH, Korea Centers for Disease Control and Prevention (KCDC), where the sera and blood were separated and stored at -20°C for future antibody and polymerase chain reaction (PCR) analyses. The study protocol was reviewed and approved by the Human Ethics Committee of the KNIH.

### Indirect fluorescent antibody test

To test for antibodies against malaria, an IFAT was performed with whole blood antigen against *P. vivax *[15-17]. Briefly, 10 ml of malaria parasite-infected blood was collected by venipuncture from *P. vivax indigenous *patients. After removing the plasma, the cells were suspended in phosphate-buffered saline (PBS, pH 7.2) and centrifuged for 5 min at 2,500 rpm. The supernatant was discarded, and the cells were resuspended in fresh PBS. The wash step was repeated three more times. Finally, an appropriate amount of PBS was added to maintain the parasitemia at no less than 1%. Cells were added to each well of Teflon coated slides. After being dried at room temperature for 12 hrs, the slides were stored at -70°C. To determine the antibody titers against *P. vivax *for each patient, the antigen slides were fixed in pre-cooled acetone (-20 °C) for 10 min and washed with PBS; then 20 μl of 1:32 to 1:8,192 (vol/vol) was diluted sera was added to each well. Positive and negative controls were spotted onto each slide and incubated in a humidified chamber for 30 min at 37°C. The reactions were stopped by washing the reacted sera with PBS. The slides were immersed in PBS for 6 min and then dried at room temperature. Diluted FITC-conjugated anti-human IgG (Sigma, 1:32 vol/vol in PBS) was added to each well, incubated, and washed using the same method described above. Several drops of buffered glycerol were added to the samples, and the slides were covered with coverslips. The slides were examined under a 40× objective of a fluorescence microscope.

### Polymerase chain reaction (PCR)

Genomic DNA was extracted from the blood samples using a QIAamp Blood Kit (Qiagen). PCR was performed with AccuPower PCR Premix (Bioneer), 50 ng of purified genomic DNA, and 40 pmol each of each forward (MSP-F; 5'-ACCATGTGTATAGACACCAATGTGCCTGATAATGCA-3') and reverse (MSP-R; 5'-TTAAAGCTCCATGCACAGGAGGAAAAGCAA-3') primer to amplify interspecies conserved block 10 (ICB10). The final volume was adjusted to 20 μl with distilled water. The cycling conditions were as follows: (1) denaturation at 94°C for 5 min, (2) 40 cycles of 1 min at 94°C, 1 min at 62°C, and 1 min at 72°C, and (3) incubation at 72°C for 5 min [18-21]. PCR products were analysed on a 1.2% agarose gel and viewed on a UV transilluminator.

### Calculation of the annual parasite index (API)

The annual parasite index (API) was calculated as the incidence of malaria per 1,000 residents.

### Data analysis

Relationships between antibody titer and PCR-positivity rate, positive rates of IFAT and age groups, and dates of blood collection and PCR-positive cases were analysed by correlation. Analysis of the annual parasite index (API) was carried out using Kruskal-Wallis test. Relationship between APIs and IFAT positivity rates of villages were analysed by Two-way ANOVA. Data analyses were performed using GraphPad (GraphPad Software, Inc., La Jolla, CA).

## Results

### Blood collection

**Figure 1:**
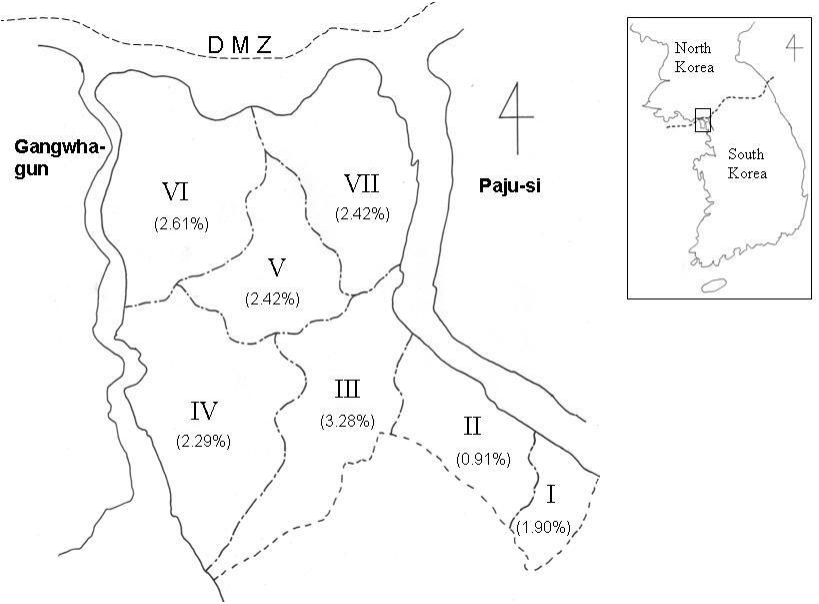
**Study areas**. I, Gochon-eup; II, Gimpo-dongs (Gimpo 1-dong, Gimpo 2-dong, Sau-dong, and Pungmu-dong); III, Yangchon-myeon; IV, Daegot-myeon; V, Tongjin-myeon; VI; Wolgot-myeon; and VII, Haseong-myeon. ( ) indicates the positivity rate of IFAT.

The location of the study area is shown on the accompanying map. Four villages, Gimpo 1-dong, Gimpo 2-dong, Sau-dong, and Pungmu-dong, are adjacent to each other and are collectively considered as Gimpo-dongs, resulting in seven sites located on the map (Figure 1). All samples were collected from seven villages located in Gimpo-si, Gyeonggi-do, South Korea. In this area, a mean of 3.92% of residents per village were sampled. A total of 5,797 blood samples were collected from the 148,066 inhabitants of Gimpo-si in 1999. Among the blood samples, 2,423 were collected from males, and 3,374 were from females, resulting in a 1:1.39 sex ratio. The mean age was 48.78 years (range 2 to 97 years).

### Positivity rate of IFAT

The criteria for a positive IFAT result were established in a previous experiment. Briefly, when 86 individuals and 58 vivax malaria patients were tested, normal individuals had a serum dilution under 1:16 (range from 0 to 1:16), and the 58 vivax malaria patients had a serum dilution over 1:256 (range from 1:256 to 1:4096). Therefore, a positive antibody response was defined against vivax malaria as a serum dilution above (≥ )1:32 [22]. A total of 125 samples from 5,797 people (2.16%) were positive by IFAT. Yangchon-myeon showed the highest positivity rate at 3.28% (9/274, API: 1.23), followed by Wolgot-myeon (2.61%, 29/1,109, API: 2.90), Tongjin-myeon (2.42%, 25/1,034, API: 0.71), Haseong-myeon (2.42%, 25/1,033, API: 2.15), Daegot-myeon (2.29%, 19/830, API: 0.52), Gochon-eup (1.90, 8/422, API: 0.34), and Gimpo-dongs (0.91%, 10/1,095, API: 0.14). The villages located in the northern part of Gimpo-si, closest to the DMZ had a higher positivity rate than those in the southern part (Table 1). Two-way ANOVA test showed significant between APIs and IFAT positive rates of villages (*P *< 0.05).

**Table 1:** Positivity rate and distribution of fluorescent antibody responses of sera according to area

Village	No. of Sera Tested	No. of Positive Sera	^a^IFAT serum dilution endpoint								Positivity rate (%)	^b^API
					
			< 1:32	≥ 1:32	1:64	1:128	1:256	1:512	1:1024	1:2048		
Gochon-eup	422	8	414	3	4	1	0	0	0	0	1.90	0.34

Gimpo-dongs	1095	10	1085	7	1	1	1	0	0	0	0.91	0.14

Yangchon-myeon	274	9	265	8	0	1	0	0	0	0	3.28	1.23

Daegot-myeon	830	19	811	12	5	1	0	1	0	0	2.29	0.52

Tongjin-myeon	1034	25	1019	9	12	2	0	2	0	0	2.42	0.71

Wolgot-myeon	1109	29	1080	10	9	3	3	2	1	1	2.61	2.90

Haseong-myeon	1033	25	1008	14	6	4	0	1	0	0	2.42	2.15

Total	5797	125	5672	63	37	13	4	6	1	1	2.16	0.68

Note.^a^IFAT; Indirect fluorescent antibody test

^b^API; Annual parasite index from 1999

* Significant between API and IFAT positive rate was analysed by Two-way ANOVA (*P *< 0.05)

The highest positivity rate according to age group was 80-89 years (5/162, 3.09%, API: 5.62), followed by 70-79 years (19/638, 2.98%, API: 2.39). The lower positivity groups were 10-19 (7/604, 1.16%, API: 0.16), 20-29 (5/389, 1.29%, API: 0.43), 30-39 (11/784, 1.40%, API: 0.33), and 90-99 years (0/3, 0%, API: 0). These results indicate that the positivity rate of IFAT increased with age groups (*P *= 0.03), furthermore similar result was observed in API analysis (P = 0.003) (Table 2). Positivity rate (2.23%) of males is higher than the positivity rate (2.10%) of females (Table 3).

**Table 2:** Positivity rate and distribution of fluorescent antibody responses of sera according to age

Age	No. of Sera Tested	No. of Positive Sera	^a^IFAT serum dilution endpoint								Positivity rate (%)	^b^API
					
			< 1:32	≥ 1:32	1:64	1:128	1:256	1:512	1:1024	1:2048		
0 - 9	299	7	292	3	4	0	0	0	0	0	2.34	0

10 - 19	604	7	597	4	3	0	0	0	0	0	1.16	0.16

20 - 29	389	5	384	2	1	0	1	1	0	0	1.29	0.43

30 - 39	784	11	773	6	4	1	0	0	0	0	1.40	0.33

40 - 49	842	23	819	10	8	3	0	2	0	0	2.73	1.04

50 - 59	912	19	893	8	8	3	0	0	0	0	2.08	1.69

60 - 69	1164	29	1135	18	5	2	1	3	0	0	2.49	1.68

70 - 79	638	19	619	11	4	2	2	0	0	0	2.98	2.39

80 - 89	162	5	157	1	0	2	0	0	1	1	3.09	5.62

90 - 99	3	0	3	0	0	0	0	0	0	0	0	0

Total	5797	125	5672	63	37	13	4	6	1	1	2.16	0.68

Note. ^a^IFAT; Indirect fluorescent antibody test

^b^API; Annual parasite index from 1999

* It shows significant correlation between age group and IFAT positivity rate of IFAT (*P *= 0.03) (except age group 90-99).

§It shows significant correlation between age group and API (*P *= 0.003) (except age group 90-99).

**Table 3:** Positivity rate and distribution of fluorescent antibody responses of sera according to sex

Sex	No. of Sera Tested	No. of Positive Sera	*IFAT serum dilution endpoint								Positivity rate (%)
				
			<1:32	≥ 1:32	1:64	1:128	1:256	1:512	1:1024	1:2048	
Male	2418	54	2364	26	17	5	3	2	1	0	2.23

Female	3379	71	3308	37	20	8	1	4	0	1	2.10

Total	5797	125	5672	63	37	13	4	6	1	1	2.16

Note. *IFAT; Indirect fluorescent antibody test

### Relationship between PCR positive and antibody positive

**Figure 2:**
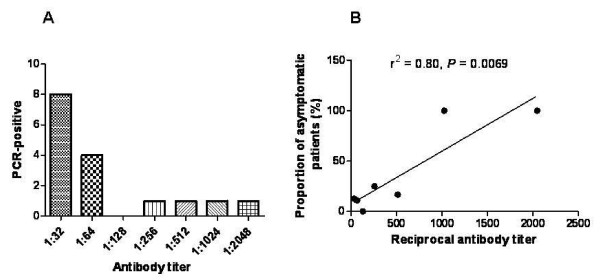
**Relationship between PCR-positive and IFAT-positive cases**. A, Number of PCR- positive cases according to antibody titer. B, Proportion of asymptomatic patients according to antibody titer.

**Figure 3:**
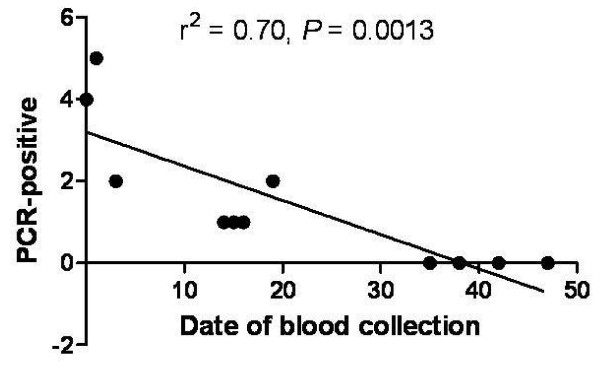
**PCR positive cases according to their collection date**.

None of the cases were positive according to microscopic examination. Therefore, genomic DNA was prepared from 125 antibody positive blood samples for PCR analysis with MSP-1 specific primers. Sixteen of the 125 (12.80%) antibody-positive cases were positive by PCR. Eight PCR-positive cases had an antibody titer of 1:32, four had a titer of 1:64, one had a titer of 1:256, one had a titer of 1:512, one had a titer of 1;1024, and one had a titer of 1:2048 (Figure 2A). The PCR positivity rate increased as the antibody titer increased (Figure 2B, *P *< 0.05). It showed a significant reduction of PCR-positive cases according to the dates of blood collection (*P *= 0.0013). Four PCR-positive cases were detected at first date of blood collection (26^th ^Oct, day 0), 5 cases at second date (27^th ^Oct, day 2), and no case at the end of blood collection (12^th ^Dec, day 47). PCR-positive cases were detected until mid-November. After that time, no additional positive cases were found. Eleven PCR-positive cases (68.75%) were collected in October, and 5 cases (31.25%) were collected in November (Figure 3). All seropositive persons were free of clinical signs or symptoms of malaria at the time of sample collection.

### Analysis of annual parasite index (API) in Gimpo-si during 1997-2006

**Figure 4:**
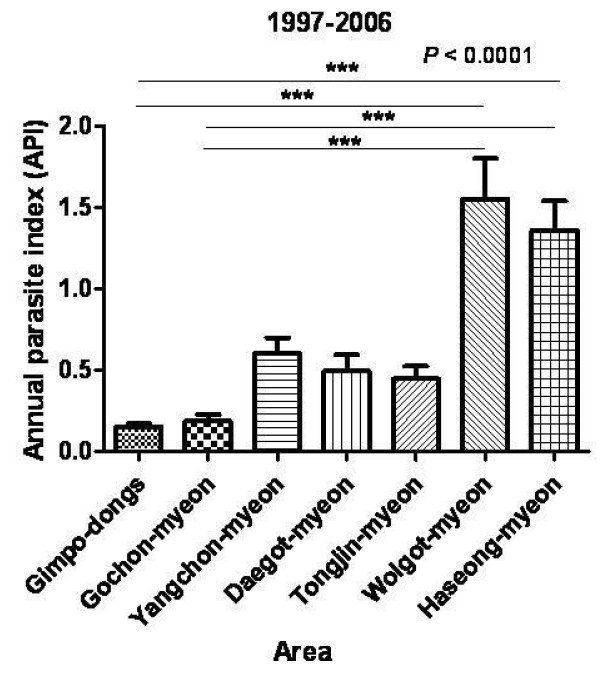
**Annual parasite index (API) according to areas from 1997 to 2006**. These values were calculated as the incidence of malaria per 1,000 residents.

**Figure 5:**
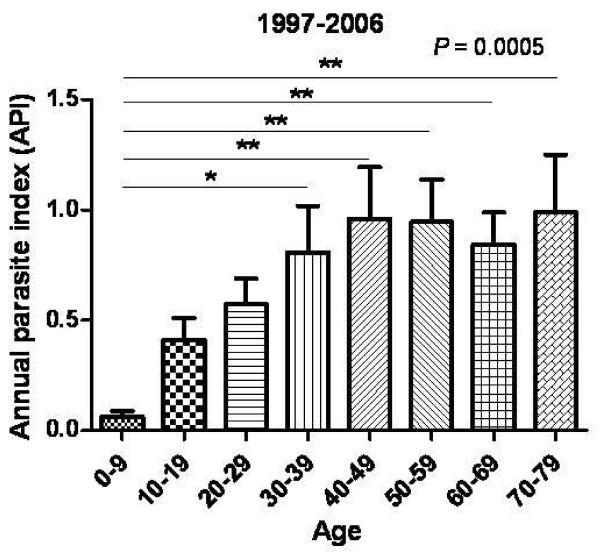
**Annual parasite index (API) according to age from 1997 to 2006**. These values were calculated as the incidence of malaria per 1,000 residents.

Annual parasitic index (API) of Gimpo-si showed 0.54 ± 0.29 during ten years (from 1997 to 2006) after reemerging vivax malaria in Gimpo-si. Among them, Wolgot-myeon (1.61 ± 0.93) showed highest API, Haseong-myeon (1.42 ± 0.65) ranked second. These areas are closed to DMZ. Yangchon-myeon (0.62 ± 0.36) ranked third. Gochon-myeon (0.19 ± 0.16) and Gimpo-dongs (0.16 ± 0.07) have a lower APIs than other areas. These areas located far from DMZ among seven study areas (Figure 4, *P *< 0.0001). APIs were increased by age groups. Age group 0-9 showed API as 0.06 ± 0.04, age group 10-19 as 0.41 ± 0.31, age group 20-29 as 0.57 ± 0.37, age group 30-39 as 0.81 ± 0.67, age group 40-49 as 0.96 ± 0.74, age group 50-59 as 0.95 ± 0.61, age group 60-69 as 0.84 ± 0.46, age group 70-79 as 0.99 ± 0.83 (Figure 5, *P *< 0.05).

## Discussion

The outbreak areas of re-emerging malaria in South Korea were mostly limited to Paju-si and Yeoncheon-gun, Gyeonggi-do, which are located within 10-15 km of the southern DMZ border [8,23]. The DMZ is a 4-km-wide and 250-km-long corridor that extends across the middle part of the Korean peninsula. No civilians have been allowed to enter into the DMZ for more than 50 years; therefore, the natural landscape ecosystems and biodiversity are highly conserved in the DMZ [9]. Each year, outbreak areas are expanding to both the south and east from DMZ and are believed to have originated from the northern part of the DMZ. Re-emerging malaria is presumed to have originated not from the immigration of infected people from the north but from mosquitoes infected with *P. vivax *that flew from the north because passage through the DMZ is almost impossible. The corridor is heavily fortified on both sides of the buffer zones with land mines and barbed wire fences [23-26].

In July and August 1997, two 15-year-old patients were reported to have indigenous vivax malaria in Gimpo-si, Gyoenggi-do. The patients were teammates on a football team at Tongjin Middle School, which is located in the north-western part of Gimpo-si, approximately 8.5 km south of the DMZ. They had no history of travelling abroad or abusing drugs [27]. A malaria team from the KNIH investigated whether vivax malaria could become prevalent in Gimpo-si in the near future. This study used IFAT because serological data can provide useful evidence for the extent and degree of malaria endemicity [11], especially in areas with low endemicity [13]. The rate of parasitaemia is the classical method for measuring the endemicity of malaria, whereas the incidence of parasitaemia alone may fail to fully provide an adequate description of the prevalence of malaria in a population. When the incidence of malaria is low, mass blood surveys do not yield results that are commensurate with the amount of work involved [28]. Therefore, the application of IFAT may reflect the prevalence of malaria in population [14].

Previously, it was observed that 16.67% (4/24) of seropositive cases exhibited malaria symptoms in the following year (authors' unpublished data). This observation suggests that some seropositive cases may be asymptomatic. Those seropositive residents that did not exhibit malaria symptoms have a history of exposure to malaria parasites. It is not clear how many seropositive cases are asymptomatic. During the winter season, malaria parasites can survive mainly but not all in the liver as hypnozoites in human. Parasites cannot survive for long periods of time in human blood due to the host immune system; therefore, they reside in the liver, which allows the parasites to evade the immune system. Park *et al *reported a similar observation. They examined the malaria status of 1,000 soldiers who served in high-risk areas, and four soldiers (0.4%) were shown to be infected with the parasite by nested PCR. They had no clinical signs or symptoms associated with *P. vivax *although one of the soldiers showed symptoms after a few weeks. These results indicate asymptomactic cases are present in the Korean population. Out of 1,713 soldiers who served in high-risk areas during *P. vivax *transmission season, 15% had antibodies for Pv200, merozoite surface protein-1 (MSP-1). Eleven of the 40 soldiers who later developed symptomatic malaria had elevated levels of anti-Pv200 antibody up to three months prior to the onset of symptoms. This result indicates that the *P. vivax *blood stage antigen may have increased during the early erythrocytic stage of infection, which indicates that the antibody test could be used in seroepidemiologic studies as an efficient tool for monitoring the prevalence of malaria in large cohorts. Considering the challenges in detecting malaria due to its long incubation period and the sometimes mild symptomatology of Korean *P. vivax*, anti-Pv200 ELISA and nested PCR could be valuable tools in a programme to systematically eradicate malaria from Korea [29].

Based on the findings mentioned above, the prevalence of malaria was investigated in a low endemicity area (Gimpo-si). 5,797 blood samples were collected from residents of Gimpo-si in 1999. 125 seropositive cases were detected, 12.80% (16 cases) of which were also positive by PCR. These results indicate that the antibody level titers obtained by IFAT may increase the likelihood of identifying asymptomatic patients. This technique will be helpful in the early eradication of malaria in Korea. Additional studies should be examine the relationship between antibody titer and PCR results, specifically whether high antibody titers obtained by IFAT increase the chances of finding PCR positive cases, as shown in Figure 2 (*P *< 0.05). PCR-positive cases were correlated with the pattern of the incidence of malaria. Patient cases were reported from March to November, with a peak in August [23,24], because any PCR- positive cases did not identify in December (Figure 3). The Yangchon-myeon showed the highest positivity rate (3.28%) by IFAT. This village is located in the middle of Gimpo-si and is surrounded by large rice fields. This environmental might provide a good habitat for malaria vectors. However, the annual parasite index (API) for Yangchon-myeon ranked third among the 7 villages (Table 1). As shown in Table 1 and Figure 4, the positivity rate of IFAT represents the API of each village. The positivity rate was relatively higher for the over-30 age group than under-30 age group (*P *< 0.05). Additionally, the positivity results for IFAT were similar to the API for 1999 (Table 2, *P *< 0.05). In addition, the APIs from 1997 to 2006 supported the observation that the positivity rate for IFAT increased with age (Figure 5, *P *< 0.05). During malaria transmission season, adults of this age often work from early in the morning to late at night in the fields without protection from mosquitoes. Children in the under-9 age group showed a high positivity rate (2.34%). The children are most likely to be exposed to malaria vectors with their parents during evening activities. However, the 10 to 39 age group showed a relatively low positivity rate; students and business workers normally belong to this age group. Therefore, this age group would be exposed less than other age groups to malaria vectors due to their lifestyles (Table 2). The positivity rate of males was higher than that of females. Males may have more outdoor activities than females (Table 3).

## Conclusions

The main finding from this study was that antibody detection using IFAT might provide information about the prevalence of malaria in certain areas. Thus, this information might help us to understand the prevalence of malaria in the past and in the future situations. Additionally, IFAT might provide useful information about *P. vivax *malaria infections in epidemic areas and could be useful in diagnosing asymptomatic patients. It is important to find asymptomatic patients to eliminate vivax malaria, and the addition of a serological method to normal surveillance methods can increase the accuracy of the interpretation about the prevalence of malaria.

## Competing interests

The authors declare that they have no competing interests.

## Authors' contributions

HWL, HHK, and WJL conceived and designed the study and participated in the research. HWL wrote the manuscript. SMH, MYP, NRK, SHC, TSI, JYK, HK, and YS collected the blood samples in the field. MYP, HK, YS, HHK, and TSI performed the IFAT and PCR assays. HWL read all IFAT slides. JS, HWL, JKL, and WJL helped to design the study. All authors have read and approved the final manuscript.
